# The Occupational Dimension of Musculoskeletal Disorders: A Comparison of Healthcare Workers and Administrative Staff Using the NMQ-E Tool

**DOI:** 10.3390/jcm14176187

**Published:** 2025-09-02

**Authors:** Magdalena Matuszewska, Łukasz Rypicz, Izabela Witczak, Anna Kołcz

**Affiliations:** 1 Division of Public Health, Wroclaw Medical University, 50-367 Wroclaw, Poland; magdalena.matuszewska@umw.edu.pl; 2 Division of Health Care Organization, Wroclaw Medical University, 50-367 Wroclaw, Poland; izabela.witczak@umw.edu.pl; 3 Independent Medical Education and Simulation Laboratory in Physiotherapy, Wroclaw Medical University, 50-367 Wroclaw, Poland; anna.kolcz@umw.edu.pl

**Keywords:** MSDs, healthcare workers, administrative staff

## Abstract

**Background**: Musculoskeletal disorders (MSDs) are a significant health problem associated with performing professional activities. The occurrence of pain often determines the type of work undertaken. Healthcare workers and public administration employees are occupational groups at risk of developing musculoskeletal disorders. The aim of this study was to compare the incidence and location of MSDs between two professional groups—healthcare and administrative workers—and to assess their impact on professional functioning. **Methods**: The study included 339 employees, comprising 188 healthcare workers and 151 administrative workers. An extended version of the Nordic Musculoskeletal Questionnaire (NMQ-E) was used. **Results**: Healthcare workers experienced MSDs significantly more often than administrative workers, both in the past and in the last 12 months. MSDs in healthcare workers more often led to absenteeism, changes in duties, use of healthcare services, and medication use. The observed differences were statistically significant, particularly in the upper and lower limbs. **Conclusions**: Musculoskeletal disorders (MSDs) are a significant health problem among healthcare and administrative workers, but they affect healthcare staff much more frequently, especially in the upper and lower limbs. This results in higher sick leave rates, the need to modify duties, and more frequent use of treatment and painkillers. These differences may result from varying working conditions, age, and education, highlighting the need to implement preventive measures tailored to the specific characteristics of each professional group.

## 1. Introduction

Musculoskeletal disorders (MSDs) are a significant health issue that can result from performing typical occupational activities. This condition affects many professional groups, including healthcare workers and administrative staff. The World Health Organization (WHO) recognizes MSDs as a pressing problem and a major challenge for healthcare managers and public health experts. This is due to the increasing prevalence of MSDs and their negative impact on the ability to perform professional duties, which, in turn, adversely affects the quality of life of economically active individuals. Considering current demographic trends—such as delayed retirement and prolonged periods of work in constrained or sedentary positions—the issue of MSDs is becoming increasingly important from an epidemiological perspective [1,2].

The etiology of MSDs in the workplace is multifactorial and includes both biomechanical and psychosocial aspects [3]. Employees who perform their professional duties in forced body positions under dynamic or static loads are particularly at risk. For healthcare workers, the main risk factors include activities such as lifting, carrying, and moving patients and medical equipment, as well as prolonged standing [4]. Among administrative workers, the predominant risk factor is prolonged sitting at computer workstations that often do not meet ergonomic requirements [5]. Additionally, the development and severity of MSDs are influenced by psychosocial factors, such as occupational stress, an excessive workload, and time pressure. These conditions can lead to increased muscle tension and chronic pain.

The occurrence of MSDs significantly affects one’s ability to work, leading to the need to modify professional duties, take more frequent sick leave, and reduce work efficiency [6]. In cases of chronic conditions, the result may be a permanent reduction in the ability to work. Studies conducted among Polish physiotherapists and nurses have shown that MSDs are associated with poorer health behaviors, including lifestyle habits that negatively affect physical and mental health, and a higher risk of developing chronic diseases [7]. Comprehensive therapeutic management includes pharmacotherapy, physiotherapy, and even surgery. This generates significant direct costs related to treatment and indirect costs resulting from reduced work capacity [8,9]. Research by Valim et al. in Brazil has shown that MSDs are strongly associated with burnout and mental distress among nurses, further exacerbating poor health and requiring a holistic therapeutic approach [10].

Despite numerous epidemiological studies, there is still a lack of comprehensive analyses examining the impact of MSDs on work capacity and the use of health services in selected occupational groups. This study aimed to evaluate the impact of MSDs on the professional functioning of healthcare and administrative workers. It assessed the significance of symptom occurrence in relation to changes in job responsibilities, sick leave frequency, commonly used treatment methods, and the association between MSD symptoms and sociodemographic or occupational factors. The results help identify differences in pain frequency and location between healthcare and administrative workers and highlight areas requiring corrective action.

## 2. Materials and Methods

The study was conducted with the following two groups of employees: one group consisted of 188 healthcare workers, and the other included 151 administrative workers. The healthcare group comprised nurses, midwives, and other medical professionals employed in regional hospitals and outpatient clinics. The administrative group consisted of individuals employed in office-based positions within public administration institutions located in the same urban area as the healthcare facilities, ensuring comparable socio-economic and geographical conditions.

The Polish version of the Nordic Musculoskeletal Questionnaire—Extended (NMQ-E) was used to assess the prevalence and characteristics of musculoskeletal disorders. The original version of the NMQ-E questionnaire was developed by Dawson et al. [11]. This tool enables a detailed evaluation of pain presence and location, its duration (e.g., past 12 months, past 4 weeks, and day of the survey), and its impact on professional functioning, including the use of healthcare services, sick leave, and changes in job responsibilities. The version of the NMQ-E used in this study was based on a previously translated and culturally adapted Polish version.

In addition to the NMQ-E, the questionnaire package included a demographic and occupational profile section, which gathered data on age, sex, body mass index (BMI, calculated from self-reported height and weight), educational level, job tenure, and job type. Data on the use of sick leave due to musculoskeletal pain were collected via a dedicated section listing specific body areas and asking whether pain in those areas had led to time off work.

The questionnaires, accompanied by an information sheet, were distributed to participants during informational meetings held at their workplaces. After completion, participants deposited the questionnaires in sealed envelopes in designated collection boxes to ensure confidentiality. The inclusion criteria included employment at the participating institutions and the provision of informed, voluntary consent to participate. The study was approved by the Bioethics Committee of the Medical University of Wrocław (KB-184/2019). All participants were informed about the study’s purpose, voluntary and anonymous nature, and their right to withdraw at any time.

The statistical analysis involved making comparisons between groups using Pearson’s chi-square test or Fisher’s exact test for qualitative variables and the Mann–Whitney test for quantitative variables. The level of statistical significance was set at *p* < 0.05. Data analysis was performed using R software, version 4.2.2.

## 3. Results

### 3.1. Characteristics of the Study Group

People under 40 made up 41.9% of the total sample. Among the administrative staff, this percentage was 49.7%, while among the healthcare workers, it was 35.6%. The percentage of participants in the 41–50 age group was similar in both groups (29.3% among healthcare workers and 31.8% among administrative staff). By contrast, people over 50 accounted for 35.1% of the healthcare worker group and 18.5% of the administrative staff group. These differences in age distribution were statistically significant (*p* = 0.002). The average age was higher among healthcare workers, which may be relevant in the context of the prevalence of chronic musculoskeletal disorders. Women constituted the vast majority of the study population: 93.6% in the healthcare worker group and 91.4% in the administrative staff group. However, the gender distribution did not differ significantly between the groups (*p* = 0.568). In contrast, the level of education differed significantly between the groups (*p* < 0.001). In the administrative staff group, 91.4% of respondents had a master’s degree. Among healthcare workers, 36.7% had a master’s degree; a significant percentage had a secondary education (33.0%) or a bachelor’s degree (30.3%). The BMI distribution was similar in both groups: 59.6% of healthcare workers and 57.6% of administrative staff reported a normal BMI. Meanwhile, 28.7% and 26.5% were overweight and 10.6% and 15.9% were obese, respectively (*p* = 0.372). Statistically significant differences were also noted in length of service between the groups (*p* < 0.001). The largest percentage of the healthcare worker group had over 25 years of service (37.2%), while the majority of the administrative staff group had between 11 and 25 years of service (60.3%). Shorter seniority of up to 10 years was reported by 34.0% of healthcare workers and 21.9% of administrative staff. Detailed data are summarized in Table 1.

### 3.2. Musculoskeletal Disorders—Comparative Results of Two Groups

The most commonly reported complaints were related to the lower back (75.8% of participants), neck (69.0%), and upper back (63.4%). Healthcare workers reported shoulder (53.2% vs. 41.7%; *p* = 0.046), elbow (29.3% vs. 11.3%; *p* < 0.001), thigh/hip (39.9% vs. 25.2%; *p* = 0.006), and ankle/foot (48.9% vs. 31.1%; *p* = 0.001) complaints significantly more often than administrative workers. No significant differences were found in the frequency of neck, upper back, wrist/hand, lower back, and knee complaints.

The age at which the first complaints appeared was similar in both groups, except for upper back pain, which appeared significantly later among healthcare workers than administrative workers (median: 37 vs. 30 years; *p* = 0.02).

There were no significant differences between the groups in the frequency of hospitalization due to musculoskeletal complaints (all *p* > 0.05).

Healthcare workers were significantly more likely than administrative workers to change jobs or job duties due to neck pain (7.5% vs. 1.3%; *p* = 0.017), upper back pain (7.5% vs. 0.7%; *p* = 0.006), elbow pain (3.2% vs. 0%; *p* = 0.035), lower back pain (12.8% vs. 0.7%; *p* < 0.001), thigh/hip pain (6.9% vs. 0%; *p* = 0.003), and knee pain (4.8% vs. 0%; *p* = 0.005).

#### 3.2.1. Frequency of Pain During the Last 12 and 4 Months, as Well as on the Day of the Examination

Over the past 12 months, healthcare workers reported significantly more complaints of pain in the following areas: shoulders (42.6% vs. 28.5%; *p* = 0.01), elbows (21.8% vs. 6.0%; *p* < 0.001), wrists/hands (37.8% vs. 26.5%; *p* = 0.037), thighs/hips (32.5% vs. 17.9%; *p* = 0.004), and ankles/feet (37.8% vs. 21.2%; *p* = 0.001). There were no significant differences in the frequency of neck, upper back, lower back, and knee pain.

However, when analyzing complaints from the past four months, healthcare workers reported pain significantly more often in the neck (59.0% vs. 45.7%; *p* = 0.019), shoulders (39.4% vs. 26.5%; *p* = 0.017), elbows (22.9% vs. 3.3%; *p* < 0.001), thighs/hips (29.8% vs. 16.6%; *p* = 0.007), and ankles/feet (36.7% vs. 19.2%; *p* = 0.001).

On the day they completed the questionnaire, healthcare workers experienced neck pain significantly more often than administrative workers did (31.4% vs. 17.2%; *p* = 0.004). They also experienced upper back pain more often (28.7% vs. 13.3%; *p* = 0.001) and pain in the elbow (11.2% vs. 0.7%; *p* < 0.001), wrists/hands (14.9% vs. 6.0%; *p* = 0.014), lower back (40.4% vs. 21.9%; *p* < 0.001), thighs/hips (18.6% vs. 8.0%; *p* = 0.008), knees (21.3% vs. 10.6%; *p* = 0.013), and ankles/feet (20.7% vs. 8.0%; *p* = 0.002).

#### 3.2.2. The Impact of MSD on Work Ability and the Need for Treatment

Over the past 12 months, musculoskeletal disorders prevented the healthcare workers from performing their daily duties significantly more often than administrative staff, particularly due to pain in the neck (28.7% vs. 13.9%; *p* = 0.002) and shoulders (19%). 7% vs. 8.6%; *p* = 0.007), upper back (26.1% vs. 10.6%; *p* = 0.001), elbows (12.2% vs. 1.3%; *p* < 0.001), wrists/hands (20.7% vs. 7.3%; *p* = 0.001), lower back (37.8% vs. 21.2%; *p* = 0.001), and thighs/hips (21.3% vs. 6.6%; *p* < 0.001).

Healthcare workers were significantly more likely than administrative workers to visit a doctor, physiotherapist, or other specialist for neck (38.8% vs. 26.5%; *p* = 0.023), shoulder (23.9% vs. 13.9%; *p* = 0.029), upper back (35.1% vs. 20.5%; *p* = 0.005), elbow (14.9% vs. 3.3%; *p* = 0.001), or lower back (41.5% vs. 29.8%; *p* = 0.035) pain.

Neck, shoulder, upper back, elbow, wrist/hand, and lower back pain required medication more often among healthcare workers than administrative workers (all *p* < 0.05).

Healthcare workers were significantly more likely to take sick leave due to shoulder (11.2% vs. 3.3%; *p* = 0.013), upper back (13.3% vs. 5.9%; *p* = 0.04), elbow (9.6% vs. 0%; *p* < 0.001), wrist/hand (11.2% vs. 2%; *p* = 0.002), lower back (17% vs. 8%; *p* = 0.021), and thigh/hip (8.5% vs. 2%; *p* = 0.018) pain.

The study showed that healthcare workers experienced musculoskeletal disorders significantly more often than administrative workers—both in terms of having “ever” experienced a disorder and experiencing one in the past 12 months, past 4 months, and on the day of the study. These complaints frequently led to changes in job duties, the use of healthcare services, medication intake, and sick leave. The most pronounced differences between the groups concerned the upper limbs (shoulders, elbows, and wrists/hands) and lower limbs (thighs/hips and ankles/feet), as well as the impact of these complaints on daily functioning (Table 2 and Table 3).

#### 3.2.3. Association Between Sociodemographic Factors and Sick Leave

Additional analysis was conducted to assess the relationship between selected sociodemographic variables and the use of sick leave due to musculoskeletal disorders. A statistically significant association was found between age and the use of sick leave, with older individuals more likely to report taking leave (*p* = 0.044). No significant association was observed for sex (*p* = 0.833). Although the difference was not statistically significant, there was a trend suggesting that individuals with higher BMI values were more likely to take sick leave (*p* = 0.083).

## 4. Discussion

The results of this study confirm that musculoskeletal disorders (MSDs) affect employees across various occupational groups. Healthcare workers experienced these disorders significantly more often than administrative workers. This finding is consistent with previous international studies on healthcare workers. One meta-analysis of European studies identified nurses as one of the occupational groups most at risk of pain, particularly in the lower back and neck areas [12,13]. The most commonly reported pain locations were the lower and upper back and neck. Data analysis showed that healthcare workers reported pain in their shoulders, elbows, thighs/hips, and ankles/feet significantly more often, from both the “ever” perspective and in the last 12 and 4 months, as well as on the day of the study. These results align with studies of nurses, doctors, and other medical professionals, which identify repetitive movements, lifting, and forced positions as primary risk factors for MSDs [3,14]. Research on intensive care unit (ICU) staff shows that the specific nature of working with patients often makes it difficult to maintain ergonomic working conditions [15]. Similar conclusions were reached in studies of nurses working in hemodialysis units, where a high incidence of MSDs was reported due to the specific requirements of the job [16]. Operating room staff also experience significant MSD-related burdens due to their professional activities [17]. Studies of dentists also confirm a high incidence of musculoskeletal disorders (MSDs) in this profession, particularly in the neck, back, and upper limbs [18]. Notably, a significant percentage of people reporting complaints was observed among administrative workers, with nearly half reporting lower back pain and over 30% reporting neck complaints in the last year. Long hours of sitting and poor workplace ergonomics pose a significant threat to the proper functioning of the musculoskeletal system. These results highlight the need for preventive measures that cover various occupational groups, regardless of their work’s nature [1]. Studies in the footwear industry show that employees in other industries are also at significant risk of MSDs, indicating the widespread and universal nature of this problem [19].

In this study, respondents over 50 years of age accounted for 35.11% versus 18.54% in the respective groups. There was also a significant difference in the level of education between the groups (*p* < 0.001). Among healthcare workers, secondary education (32.98%) and bachelor’s degrees (30.32%) were most common, while the majority (91.39%) of the administrative group had master’s degrees. The analysis considered the impact of age and level of education on the incidence of musculoskeletal disorders. Numerous publications indicate that age is one of the main risk factors for developing chronic musculoskeletal disorders [3,6]. With age, bone and joint structures naturally degenerate and tissue elasticity decreases, contributing to overload and microtrauma [20]. A higher level of education, on the other hand, may be associated with greater health awareness and care, as confirmed by studies on healthcare workers [21]. The differences in education level between the study groups may partly explain the observed differences in reported complaints. However, verification of this hypothesis would require additional analyses that were beyond the scope of this study.

The results of the study show no significant differences in the incidence of neck and lower back pain between groups when viewed over time, but on the day of the study, these differences were significant. Similar results have been obtained by other researchers, indicating that lumbar spine pain occurs with a similar frequency among manual and office workers but has different causes and risk factors [6]. Healthcare workers predominantly experience sudden, acute pain related to overexertion, while administrative workers predominantly experience chronic pain resulting from prolonged incorrect posture during work [2].

In the future, it would be worthwhile to expand the scope of the analysis to include psychosocial factors. Psychosocial factors, such as stress and lack of support, are considered significant in exacerbating MSD symptoms, so it is difficult not to refer to them. Support for these phenomena can also be found in the literature [3,10]. Research conducted by Valim and colleagues in Brazil showed that MSDs are strongly associated with burnout and mental distress [10], and similar results were reported in studies by Buunaaisie and colleagues among nurses and midwives, where MSD prevalence exceeded 80% [22]. Meta-analyses of the impact of occupational stress on lower back pain also confirm this phenomenon, showing that psychosocial factors—such as lack of social support—are significantly associated with the severity of pain symptoms [3,23].

Musculoskeletal pain often led to sick leave, a change in job responsibilities, and the use of medical assistance among healthcare workers. Employees in this group more often visited specialists, took painkillers, and took sick leave. These data are consistent with international observations, in which musculoskeletal disorders (MSDs) are one of the main causes of sick leave among healthcare workers [6,13]. Detailed analyses of selected professional groups (e.g., nurses) show that chronic musculoskeletal pain is the main cause of work absenteeism. It also leads to a decreased quality of patient care and increased staff turnover [20]. Treating MSDs among healthcare workers accounts for a significant percentage of total healthcare expenditures. The indirect costs associated with lost working capacity, organizing replacements, and increased staff turnover are even higher [8].

The results of research on the effectiveness of preventive measures are worth implementing. For example, an exercise program for nurses involved in hemodialysis showed a significant reduction in pain intensity and improved functionality. Similarly, comprehensive prevention programs implemented in Vietnamese hospitals significantly reduced the incidence of MSDs among nursing staff [24,25]. Studies conducted in Poland also indicate a significant relationship between health behaviors and MSD incidence [7].

However, this study has certain limitations, including its cross-sectional design and reliance on self-assessment by participants, which may lead to under- or over-reporting of symptoms. Nevertheless, the results are consistent with other authors’ observations and represent an important contribution to our understanding of MSDs among workers in various occupational groups. While we did not directly examine the impact of staff shortages and increasing administrative requirements, the literature suggests that these factors may exacerbate the severity of musculoskeletal disorders among healthcare and administrative workers.

The findings confirm that musculoskeletal disorders (MSDs) are a growing health concern across various occupational groups. The results can also serve as a basis for implementing comprehensive prevention programs. The most effective measures combine education in ergonomics, modification of the work environment, and regular physical exercise [26]. Effective prevention programs should be long-term and multifaceted, considering both physical and psychosocial factors. Using new technologies, such as real-time body position monitoring systems and regular position change reminders, shows promise [5]. The consistent application of ergonomic principles in daily professional practice, among both healthcare and administrative workers, may contribute to reducing the burden of MSDs in the workplace.

## 5. Conclusions

Musculoskeletal disorders (MSDs) are a significant health problem among healthcare and administrative workers, affecting their ability to perform professional duties and access healthcare services.

Healthcare workers experience MSDs significantly more often than administrative workers, especially in the upper (shoulders, elbows, and wrists) and lower (thighs, hips, ankles, and feet) limbs. This results in a higher rate of sick leave, a need to change job responsibilities, and more frequent use of treatment and pain medication.

The variation in the incidence and consequences of MSDs among different occupational groups may be related to various factors, including working conditions, age, and education level.

Implementing preventive measures tailored to the specific nature of the work performed by both groups is advisable. These measures should take into account workplace ergonomics, employee education, and psychosocial support.

Introducing systematic screening, ergonomic audits, and promoting physical activity may reduce the negative impact of MSDs on employees’ health and professional effectiveness.

Since this study did not directly analyze the impact of organizational and psychosocial factors, further research is necessary to better understand and counteract the occurrence of musculoskeletal disorders.

## Figures and Tables

**Table 1:** Characteristics of the study group.

Parameter	Healthcare Workers (*n* = 188)	Administrative Staff (*n* = 151)	Total (*n* = 339)	*p*-Value *
**Sex**				0.568
Female	176 (93.62%)	138 (91.39%)	314 (92.63%)	
Male	12 (6.38%)	13 (8.61%)	25 (7.37%)	
**Age**				0.002 *
≤40 years	67 (35.64%)	75 (49.67%)	142 (41.89%)	
41–50 years	55 (29.26%)	48 (31.79%)	103 (30.38%)	
>50 years	66 (35.11%)	28 (18.54%)	94 (27.73%)	
**BMI**				0.372
Normal	112 (59.57%)	87 (57.62%)	199 (58.70%)	
Overweight	54 (28.72%)	40 (26.49%)	94 (27.73%)	
Obese	20 (10.64%)	24 (15.89%)	44 (12.98%)	
No data available	2 (1.06%)	0 (0.00%)	2 (0.59%)	
**Education**				<0.001 *
Secondary (high school)	62 (32.98%)	2 (1.32%)	64 (18.88%)	
Bachelor’s degree	57 (30.32%)	9 (5.96%)	66 (19.47%)	
Master’s degree	69 (36.70%)	138 (91.39%)	207 (61.06%)	
No data available	0 (0.00%)	2 (1.32%)	2 (0.59%)	
**Work experience**				<0.001 *
≤10 years	64 (34.04%)	33 (21.85%)	97 (28.61%)	
11–25 years	52 (27.66%)	91 (60.26%)	143 (42.18%)	
>25 years	70 (37.23%)	25 (16.56%)	95 (28.02%)	
No data available	2 (1.06%)	2 (1.32%)	4 (1.18%)	

* Statistically significant.

**Table 2:** Consequences of musculoskeletal disorders—comparison of study groups.

	Healthcare Workers (*n* = 188)	Administrative Staff (*n* = 151)	*p*-Value *
**Seek medical help**	88 (46.81%)	56 (37.09%)	**0.019**
**Take painkillers**	91 (48.40%)	56 (37.09%)	**0.029**
**Sick leave**	53 (28.19%)	16 (10.60%)	**<0.001**
**Change job duties**	41 (21.81%)	11 (7.28%)	**<0.001**
**Change job position**	26 (13.83%)	2 (1.32%)	**<0.001**

* Statistically significant *p* < 0.05.

**Table 3:** Change in job position due to location of pain.

Location of Pain	Healthcare Workers (*n* = 188)	Administrative Staff (*n* = 151)	Total (*n* = 339)	*p*-Value *
**Neck**	14 (7.45%)	2 (1.32%)	16 (4.72%)	**0.017**
**Upper back**	14 (7.45%)	1 (0.66%)	15 (4.42%)	**0.006**
**Elbows**	6 (3.19%)	0 (0.00%)	6 (1.77%)	**0.035**
**Lower back**	24 (12.77%)	1 (0.66%)	25 (7.37%)	**<0.001**
**Thighs/hips**	13 (6.91%)	0 (0.00%)	13 (3.83%)	**0.003**
**Knees**	9 (4.79%)	0 (0.00%)	9 (2.65%)	**0.005**

* Statistically significant *p* < 0.05.

## Data Availability

Data available from the corresponding author.
